# Research on Tbps and Kilometer-Range Transmission of Terahertz Signals

**DOI:** 10.3390/mi16070828

**Published:** 2025-07-20

**Authors:** Jianjun Yu, Jiali Chen

**Affiliations:** Key Laboratory for Information Science of Electromagnetic Waves (MoE), Fudan University, Shanghai 200433, China; 23210720141@m.fudan.edu.cn

**Keywords:** terahertz communication, long-distance transmission, atmospheric absorption, photonic-assisted, polarization multiplexing, maximal ratio combining, high-gain antennas, power amplifiers, coherent detection, satellite–ground communication

## Abstract

THz communication stands as a pivotal technology for 6G networks, designed to address the critical challenge of data demands surpassing current microwave and millimeter-wave (mmWave) capabilities. However, realizing Tbps and kilometer-range transmission confronts the “dual attenuation dilemma” comprising severe free-space path loss (FSPL) (>120 dB/km) and atmospheric absorption. This review comprehensively summarizes our group′s advancements in overcoming fundamental challenges of long-distance THz communication. Through systematic photonic–electronic co-optimization, we report key enabling technologies including photonically assisted THz signal generation, polarization-multiplexed multiple-input multiple-output (MIMO) systems with maximal ratio combining (MRC), high-gain antenna–lens configurations, and InP amplifier systems for complex weather resilience. Critical experimental milestones encompass record-breaking 1.0488 Tbps throughput using probabilistically shaped 64QAM (PS-64QAM) in the 330–500 GHz band; 30.2 km D-band transmission (18 Gbps with 543.6 Gbps·km capacity–distance product); a 3 km fog-penetrating link at 312 GHz; and high-sensitivity SIMO-validated 100 Gbps satellite-terrestrial communication beyond 36,000 km. These findings demonstrate THz communication′s viability for 6G networks requiring extreme-capacity backhaul and ultra-long-haul connectivity.

## 1. Introduction

THz communication stands as a core candidate technology for next-generation wireless communication. Its necessity stems from the fundamental contradiction between the exponential growth in wireless data rates and the limitations of existing spectrum resources [1,2,3,4,5]. As 5G mmWave bands gradually become saturated, traditional microwave bands (<6 GHz) face inherent drawbacks including excessively wide beams, high power consumption, and insufficient bandwidth, pushing their spectral efficiency near the theoretical limit [6,7,8,9,10]. Furthermore, their practical deployment is hindered by large device size and low integration density. While laser communication offers high bandwidth potential, its practical implementation is severely constrained by difficulties in aligning its extremely narrow beams and its high sensitivity to adverse weather conditions [11,12]. Occupying the frequency gap between microwaves and optical bands, the THz band bridges this technological void with its unique physical properties, becoming crucial for future 6G developments and beyond [13,14]. The exponential increase in wireless data rates, coupled with the inability of mega-bit-per-second microwave speeds to meet the demands of massive data transmission, has driven exploration and research into the THz band with its rich spectrum resources [15,16,17]. Its enormous contiguous bandwidth lays the physical layer foundation for building 6G networks with high data rates, low latency, and high-density ubiquitous connectivity, enabling potential speeds reaching gigabits per second (Gbps) or even terabits per second (Tbps) [18,19,20].

The sub-terahertz band (typically 0.1–0.3 THz), as standardized in ITU-R V.431 [21] and IEEE 802.15.3d [22], utilizes solid-state electronics such as Complementary Metal Oxide Semiconductors (CMOSs) and SiGe Heterojunction Bipolar Transistors (HBTs) for integrated circuit implementation to support moderate-bandwidth communications. In contrast, the multi-terahertz band (3–10 THz) falls within the ‘optically-oriented spectrum’ per IRMMW-THz Conference definitions, where quantum cascade lasers (QCLs) and photoconductive antennas dominate, enabling high-resolution spectroscopy despite severe molecular absorption losses. Bridging these extremes, the 0.3–3 THz ‘gap’ predominantly employs hybrid approaches combining Schottky diode multipliers with photo-mixers [23,24,25,26,27,28,29,30].

THz communication emerges as a technology capable of breaking the performance limits of next-generation wireless networks due to its distinct advantages. Spectrum resources: the THz band offers hundreds of GHz of contiguous bandwidth, enabling single-link Tbps-level transmission without complex spectral efficiency enhancement techniques, bridging the technological gap between mmWave and Free-Space Optical (FSO) communication. Physical properties: its short wavelength not only suppresses multi-path interference and eavesdropping risks but also supports ultra-highly directional beams, exhibiting superior stability compared to FSO in complex environments [31,32,33,34,35,36,37].

**Figure 1:**
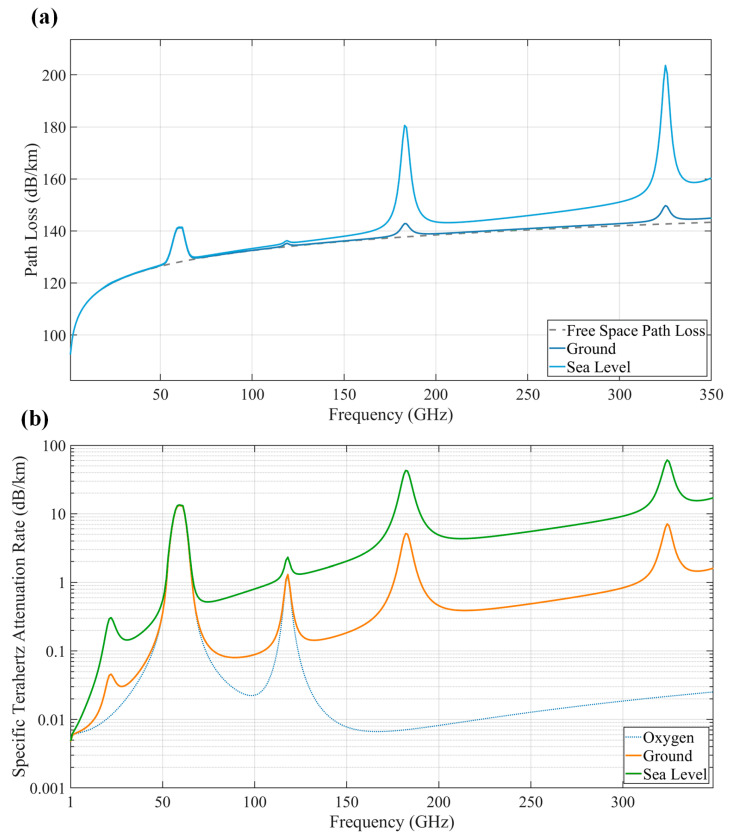
Path loss and atmospheric loss: (**a**) free space path loss; (**b**) atmospheric loss.

However, achieving long-distance THz signal transmission faces significant challenges. As indicated by Figure 1, FSPL increases exponentially with frequency; THz signals experience path loss exceeding 120 dB per kilometer, far surpassing attenuation levels in mmWave bands. Simultaneously, atmospheric attenuation effects are particularly severe in specific frequency regions: molecular resonance absorption by water vapor (H_2_O) and oxygen (O_2_) creates multiple attenuation peaks, where attenuation at sea level can exceed 10 dB/km. Under rain or fog conditions, this loss can surge to tens of dB/km. The combined effect of these two physical limitations creates a “dual attenuation dilemma” for long-distance THz transmission: signals must overcome both energy dissipation due to free-space spreading and avoid strong absorption bands outside atmospheric windows [33,38,39,40,41,42]. The engineering exploration of THz communication technology began in the 1970s. First, in 1970, Bell Labs developed the first continuous-wave THz laser (methanol laser, 1–5 THz) using gas laser pumping technology. Although constrained by cryogenic operation and low efficiency, this breakthrough marked the inception of artificially controllable THz signal generation. Subsequently, Nippon Telegraph and Telephone Corporation (NTT) achieved the world′s first long-distance THz communication experiment in 2006, demonstrating 10 Gbps over 15 km line-of-sight (LoS) using the 120 GHz band, validating THz technology feasibility for satellite backhaul and wide-area coverage scenarios. Further, during the 2008 Beijing Olympics, NTT deployed a 120 GHz THz communication system to successfully achieve real-time broadcast of 8K ultra-high-definition video signals, proving its commercial potential for applications with extreme bandwidth demands for the first time. Finally, in 2017, the IEEE published the first international THz communication standard (802.15.3d), specifying the physical layer protocol and frame structure for the 252–325 GHz band, laying the groundwork for chip design, device manufacturing, and the 6G standardization process [22,43,44,45].

This paper summarizes a series of advanced methods and architectures that achieved historic, record-breaking ultra-broadband THz signal transmissions, providing critical technological support and spectrum expansion for the transition from 5G-Advanced to 6G. In Section 2, we discuss the generation of broadband THz signals using simple and cost-effective schemes. Section 3 summarizes key enabling technologies comprehensively considering power consumption, equalization, algorithms, and structure to increase the transmission distance of THz communication systems. To explore the role of THz systems within the integrated Space–Air–Ground (SAG) strategy, Section 4 presents simulations of satellite-to-ground communications. The final section provides a summary of the paper and outlines predictions and plans for the next phase of long-distance THz communication system development.

## 2. Terahertz Signal

**Figure 2:**
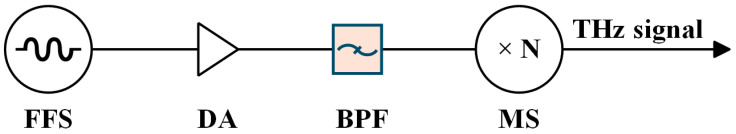
Multiplier-based terahertz signal; FFS: Fundamental Frequency Source; DA: Drive Amplifier; BPF: Band Pass Filter; MS: Multiplier Stage.

**Figure 3:**
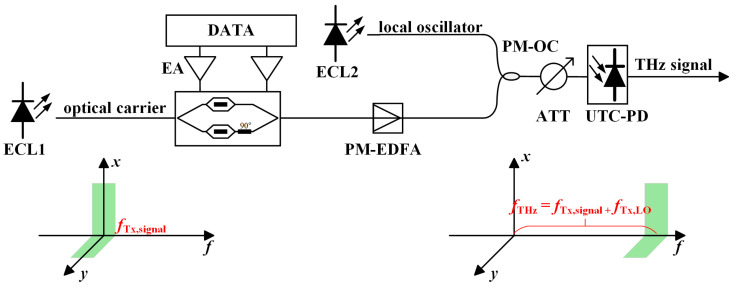
Photonic-assisted terahertz signal; ECL: external cavity laser; EA: electrical amplifier; UTC-PD: Uni-Traveling-Carrier Photodiode; PM-EDFA: polarization-maintaining erbium-doped fiber amplifier; PM-OC: polarization-maintaining optical coupler; ATT: attenuator.

Realizing an efficient, stable, and high-power THz signal source is key to the development of related applications. Especially in scenarios pursuing long-distance, high-capacity wireless communications, the performance of the signal source directly determines the system′s link budget and transmission capabilities [46,47]. Currently, as shown in Figure 2 and Figure 3, the generation of THz signals primarily relies on two main technological pathways: electronic approaches and photonic approaches.

### 2.1. Electronic Approaches

Electronic approaches primarily rely on silicon-based devices [48]. In recent years, significant progress has been made in technologies based on advanced semiconductor materials such as III-V compound semiconductors with Indium Phosphide (InP) and Gallium Nitride (GaN), including High Electron Mobility Transistors (HEMTs), Monolithic Millimeter-Wave Integrated Circuits (MMICs), and HBTs. For instance, HEMTs exhibit ultra-high electron saturation velocity and cut-off frequencies reaching 1.5 THz, laying the hardware foundation for THz communication systems. The core implementation path for all-solid-state-electronic solutions is the cascaded frequency multiplication technique [49,50,51]. An oscillator first generates a microwave/millimeter-wave fundamental frequency signal. This signal is then progressively up-converted into the THz band through frequency multiplier chains based on Schottky diodes or HBTs. Finally, an HEMT power amplifier (PA) outputs the effective radiated power [52,53,54,55,56].

**Figure 4:**
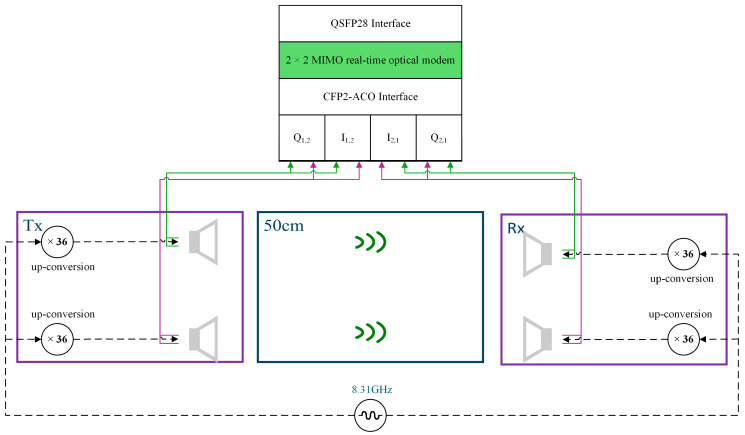
Pure-electronic terahertz real-time transmission system; Tx: transmission; Rx: reception.

In paper [57], researchers successfully demonstrated real-time transmission of 34-GBd polarization division multiplexing (PDM) quadrature phase shift keying (QPSK) signals using a purely electronic THz system integrated with a commercial Digital Coherent Optical (DCO) real-time modem. The experimental setup is shown in Figure 4. THz signal generation was achieved via electronic frequency multiplication and direct conversion mixing techniques: First, a signal generator produced an 8.311 GHz local oscillator (LO) signal, which was multiplied to ~300 GHz using a 36-fold frequency multiplier. Subsequently, the four analog baseband signals (corresponding to the X_I_/X_Q_/Y_I_/Y_Q_ components of the 34-GBd PDM-QPSK optical signal) demodulated from the first fiber span were directly fed into two MMIC-based direct-conversion in-phase and quadrature (I/Q) mixer transmitter frontends. Here, the baseband signals were up-converted to the THz band by mixing with the 300 GHz LO. Spatial multiplexing technology (using two independent antenna pairs) transmitted the dual-polarization data synchronously over a 50 cm wireless link. This method achieved the first real-time hybrid transmission with a net data rate of 100 Gb/s: Within a system comprising two fiber spans (total length 103 km) and the 300 GHz THz link, the Bit Error Rate (BER) remained stably below the forward error correction (FEC) threshold (3.4 × 10^−2^) by employing single-carrier real-time Digital Signal Processing (DSP) to jointly compensate for accumulated dispersion, polarization rotation, and phase noise. This validates the transparency of the THz wireless extender to linear impairments inherent in optical fibers and provides a multi-hundred-gigabit wireless fiber extension solution for 5G/6G backhaul.

### 2.2. Photonic Approaches

Photonic approaches fundamentally employ the dual-laser optical heterodyne beating technique for high-speed signal generation [58,59,60,61]. This technology leverages an opto-electro-optical seamless bridging architecture for signal generation and transmission: Two independent continuous-wave (CW) optical waves—one serving as the LO and the other as the modulated optical carrier—are generated by lasers. They are combined in an optical coupler for heterodyne beating, producing a THz signal whose frequency equals the difference between the two optical wavelengths. A high-speed photodetector then performs the optical-to-electrical (O/E) conversion, and the resulting THz signal is radiated by an antenna. At the receiver, a reverse conversion process reconstructs the optical carrier from the THz signal, which is finally received by a coherent receiver. This Fiber–Wireless (FiWi) integrated access system effectively combines the bandwidth and long-distance transmission advantages of optical fiber communications with the mobility and seamless coverage benefits of wireless communications. By utilizing the relatively simple and cost-effective photonically assisted THz technology, it overcomes the bandwidth bottleneck inherent in electronic devices to generate ultra-high-speed wireless THz signals.

**Figure 5:**
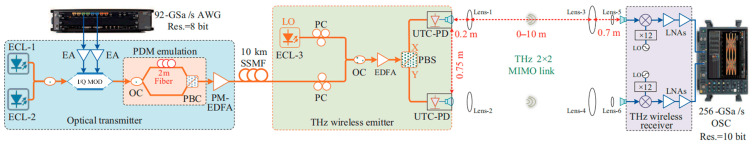
Photonic-assisted terahertz 1 Tbps transmission system; LNA: low-noise amplifier; OC: optical coupler; PC: polarization controller [62].

Prior to reference [62], real-time fiber-THz-fiber link transmissions exceeding 100 Gbit/s at frequencies above 350 GHz remained unreported. This gap stemmed from the immense challenges associated with achieving high-speed, stable, end-to-end real-time transmission at such high frequencies, including the generation, modulation, transmission loss, and real-time signal processing of high-frequency signals. Addressing this challenge, through the experimental setup in Figure 5, we successfully constructed and demonstrated, for the first time, a photonically assisted THz wireless transmission system operating in the 330–500 GHz band achieving an unprecedented Aggregated Interface User Rate (AIUR) of 1.0488 Tbps, alongside seamless integration of 10 km of fiber and a 10 m wireless link.

The core innovation lies in fully leveraging the unique advantages of photonics: The system employs free-running tunable ECLs as high-purity, low-phase-noise optical carriers (ECL-1 @ 193.47 THz, ECL-2 @ 193.565 THz). A high-speed optical I/Q modulator (Fujitsu FTM7961EX/301, 3 dB bandwidth > 35 GHz) efficiently modulates the 46 GBaud PS-64QAM baseband signal directly in the optical domain. PDM enables parallel transmission of dual-polarization signals within a single fiber. After 10 km of Standard Single-Mode Fiber (SSMF) transmission, a third ECL (ECL-3 @ 193.1 THz) serves as the LO at the receiver for optical heterodyne beating with the incoming signal. The critical O/E conversion is performed by a high-performance UTC-PD (NTT IOD-PMAN-13001). This device features an ultra-wide response bandwidth (300–2500 GHz typical), high saturation power (up to 15 dBm optical input), and good responsivity (0.15 A/W @ 1550 nm), enabling efficient, linear conversion of the heterodyne-generated difference frequency signal into powerful THz radiation. This photonically assisted THz generation method overcomes the bandwidth constraints faced by purely electronic approaches (>300 GHz), offering superior frequency flexibility, tunability, and potential bandwidth.

Finally, employing a meticulously designed 2 × 2 MIMO wireless link and an innovative real-time signal processing algorithm based on a Vector Quantized Variational Autoencoder (VQ-VAE)—specifically addressing the limitations of conventional equalizers for high-order PS-QAM signals—we successfully achieved a landmark AIUR of 1.0488 Tbps with a net rate of 828 Gbps. This groundbreaking result powerfully demonstrates the core value and immense potential of photonic technology in constructing future 6G networks demanding Tbps-level extreme connectivity.

## 3. Enabling Technologies for THz Communication Systems

In this section, to enhance the effective transmission distance of photonic-assisted THz communication systems, we introduce five key enabling technologies, specifically, improving receiver sensitivity; adopting low-order modulation formats characterized by large constellation point spacing and strong noise immunity; leveraging MIMO architectures and MIMO diversity gain for polarization-multiplexed optical THz signals; utilizing high-gain antennas and high-gain lenses; and employing PAs with high gain and high saturated output power coupled with a heterodyne coherent detection configuration at the receiver. Through this optoelectronic co-design optimization strategy, which simultaneously reduces the signal baud rate and the bandwidth requirements of the devices, we provide a feasible path for the long-distance deployment of photonic-assisted THz communications [63].

### 3.1. Selecting Appropriate Modulation Formats

**Figure 6:**
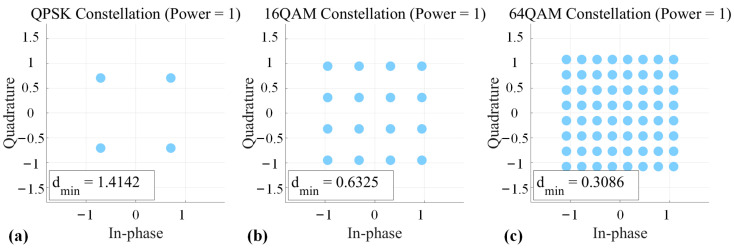
QPSK, 16QAM, and 64QAM constellation diagrams under normalized power: (**a**) QPSK; (**b**) 16QAM; (**c**) 64QAM.

In THz communication systems, achieving both long wireless transmission distance and high spectral efficiency presents a fundamental trade-off. While utilizing higher-order QAM signals rationally when extending transmission distance can yield extremely high spectral efficiency, the inherent burdens on the THz system cannot be ignored. For the same transmit power, higher-order QAM signals exhibit a smaller Euclidean distance between adjacent constellation points. Figure 6 shows the constellation diagrams of QPSK, 16QAM, and 64QAM under normalized power. Consequently, they require either higher receiver sensitivity, increased input power, or a higher signal-to-noise ratio (SNR) at the receiver side [64,65,66,67,68,69,70,71]. Furthermore, these signals are significantly constrained by the THz channel′s path loss and nonlinear effects, necessitating the integration of advanced DSP techniques and system optimization strategies [19,72,73,74].

Given our primary focus on achieving longer wireless transmission distances, we predominantly employ lower-order QAM formats, such as QPSK and 16QAM. Using 16QAM modulation, we achieved 920 Gbps·km, and using QPSK, we demonstrated transmissions spanning 30 km in the sub-THz band [75,76]. While higher-order modulation significantly boosts spectral efficiency by carrying more bits per symbol, its application is typically restricted to short-range scenarios for achieving ultra-high data rates due to the limiting characteristics of the THz channel.

### 3.2. Adopting MIMO Architecture Combining Polarization Multiplexing and MRC

In long-distance THz wireless communication systems, single-antenna architectures face severe challenges. Limitations in optoelectronic conversion efficiency cause significant energy loss during signal transmission [77]. Concurrently, the inherently poor penetration characteristics of high-frequency electromagnetic waves and severe atmospheric attenuation collectively result in extremely weak signal strength at the receiver, critically degraded SNR, and fundamental constraints on communication quality and transmission capacity [78,79,80].

**Figure 7:**
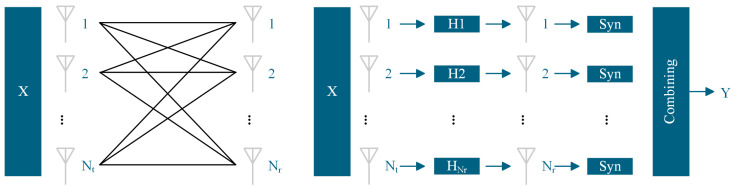
MIMO architecture diagram and MIMO diversity reception architecture.

To overcome these bottlenecks and dramatically enhance system performance, MIMO technology emerges as a critical solution. As shown in Figure 7, by deploying antenna arrays at both the transmitter and receiver ends, MIMO technology enables deep spatial exploitation, effectively addressing insufficient received power and low SNR issues [81,82]. Our primary enabling pathways include: MIMO combined with PDM (MIMO-PDM) and MIMO with MRC (MIMO-MRC) [83,84,85,86,87].

For MIMO-PDM technology, THz signals are first generated using the photonic-assisted approach introduced in Section 2, employing two optical THz signals sharing the same optical frequency but possessing orthogonal polarization states. These two signals can be generated from either (a) a common CW laser source, subsequently combined via an OC, or (b) a PDM I/Q modulator, which directly produces orthogonally polarized optical THz signals.

Prior to the photodetection (optical-to-electrical conversion) of the polarization-multiplexed optical signal, its orthogonal polarization components must be separated using optical components. Separation options include schemes based on optical couplers combined with polarization beam splitters (PBSs) or, alternatively, using a polarization-diverse phase-diversity 90-degree optical hybrid. So first, the data is modulated onto one optical carrier wave. After passing through the PBS, it beats with another LO light source (also passed through a PBS) to generate a terahertz signal.

Subsequently, in the detection stage, the dual-diode balanced structure within balanced photodetectors (BPDs) effectively suppresses common-mode noise while enhancing tolerance to input signal power and improving SNR. After passing through two high-speed balanced photodetectors, two electrical THz signals are produced, collectively forming the polarization-division-multiplexed electrical THz signal. Wireless transmission of this signal is realized via a 2 × 2 MIMO link.

At the receiver, the received THz signal must first undergo analog down-conversion. This is achieved using balanced mixers driven by an electrical LO operating in the THz frequency band, converting the THz carrier signal down to a lower intermediate frequency (IF) signal. These IF signals are then captured by a real-time oscilloscope equipped with two input ports.

Finally, the data recorded by the real-time oscilloscope is fed into an advanced DSP module for analysis and processing to recover the original information. The optical baseband transmitter generates the original optical baseband signal, whose X- and Y-polarization components can be represented as the following column vector:

(1) $$E_{in}=\left(\begin{array}{l} E_{in,x}\\ E_{in,y} \end{array}\right).$$

The received signal after fiber transmission can be represented as follows:

(2) $$E_{out1}=JE_{in}=\left(\begin{array}{cc} J_{xx} & J_{xy}\\ J_{yx} & J_{yy} \end{array}\right)\left(\begin{array}{l} E_{in,x}\\ E_{in,y} \end{array}\right).$$

where **J** is the 2 × 2 Jones matrix; the diagonal elements (*J_xx_, J_yy_*) are principal polarization channels; and off-diagonal elements (*J_xy_, J_yx_*) are the polarization crosstalk from fiber impairments.

At the Rx, the received THz signal is

(3) $$E_{out2}=GE_{out1}\cos(\omega t)=\left(\begin{array}{cc} G_{xx} & G_{xy}\\ G_{yx} & G_{yy} \end{array}\right)E_{out1}\cos(\omega t).$$

where **G** is the 2 × 2 MIMO gain matrix; ω is THz carrier frequency; and off-diagonal elements (*W_xy_, W_yx_*) are wireless crosstalk.

Combining fiber and wireless stages,

(4) $$E_{out2}=GJE_{in}\cos(\omega t)=HE_{in}\cos(\omega t).$$

where the composite channel matrix **H** is

(5) $$\begin{array}{l} H=GJ=\left(\begin{array}{cc} H_{xx} & H_{xy}\\ H_{yx} & H_{yy} \end{array}\right),\\ H_{ij}=\sum_{k=1}^{2}G_{ik}J_{kj}\;\;\;\;(i,j\in \{x,y\}). \end{array}$$

The received signals are demultiplexed via a butterfly equalizer.

**Figure 8:**
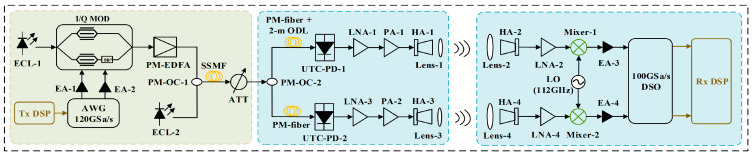
MIMO-PDM-based long-distance radio-over-fiber (RoF) terahertz transmission system; HA: horn antenna [88].

In reference [88], we successfully achieved a major breakthrough in high-speed long-distance wireless transmission in the D-band using MIMO-PDM, innovatively constructing a photonics-assisted 2 × 2 MIMO wireless transmission system. The experimental setup is shown in Figure 8. At the transmitter, dual external cavity lasers (ECL-1 @1550 nm, ECL-2 @1551 nm) provide the optical carrier and local oscillator, respectively; a 25-Gbaud 16-QAM baseband signal (generated by an arbitrary waveform generator (AWG) and amplified by an EA) is loaded via an I/Q modulator; and PDM is used to generate two independent signals. Critical opto-electronic conversion is performed by a high-performance UTC-PD, up-converting the optical signal to a 125 GHz D-band millimeter wave. Subsequently, the signal is amplified by cascaded LNAs and PAs (total gain ~31 dB, output power 13 dBm), transmitted through a pair of orthogonally polarized (horizontal/vertical) horn antennas (gain 25 dBi), and innovatively collimated and focused using large-aperture (60 cm) and small-aperture (10 cm) plano-convex lens assemblies to overcome high free-space transmission losses in the D-band. The receiver similarly employs orthogonally polarized horn antennas and lens assemblies to capture the signal, which is amplified by LNAs, down-converted to a 13 GHz intermediate frequency, and acquired by a high-speed oscilloscope. The receiver-side DSP employs a sophisticated algorithm chain including MIMO–constant modulus algorithm (CMA) equalization, frequency offset estimation, carrier phase recovery, advanced 51-tap MIMO-CMA equalization, a 183-core second-order MIMO Volterra nonlinear equalizer (MIMO-VNLE), and 155-tap decision-directed least mean square (DD-LMS) equalization, effectively mitigating impairments induced by long-distance transmission. Ultimately, over a 4.6 km real-world urban wireless link (between campuses of Fudan University), a total transmission rate of 200 Gbps (100 Gbps per channel) was successfully achieved, with a measured minimum BER of 2.05 × 10^−2^ meeting the 20% soft-decision forward error correction (SD-FEC) threshold requirement. This accomplishment established a record-breaking capacity–distance product of 920 Gbps·km.

In single-polarization MIMO systems, multiple transmitter antennas simultaneously transmit identical signals. The predominant challenge in this configuration is maximizing gain while simultaneously improving receiver accuracy (i.e., signal fidelity). MIMO-MRC addresses this by applying beamforming (BF) principles. It determines the weights at the receiver side based on the received power level of each distinct channel. Subsequently, it combines and sums the signals from all channels using these weighted coefficients to achieve diversity gain. For a MIMO system with t transmit antennas and r receive antennas, the received signal Y can be expressed as

(6) Y=HX+n.

where **X** is the *N_t_* × 1 transmit vector, **H** is the *N_r_* × *N_t_* THz channel matrix, and **n** is the *N_r_* × 1 additive white Gaussian noise (AWGN) vector satisfying E{**nn**^H^} = *N_0_*I. At the receiver, the MRC algorithm is employed, and the receiver applies a complex weight vector $W={\left[w_{1},w_{2},\ldots ,w_{N_{r}}\right]}^{T}(N_{r}\times 1)$ to the received signals and combines them:

(7) $$W^{H}Y=W^{H}HX+W^{H}n.$$

The SNR after MRC combining is derived as

(8) $$SNR=\frac{{\left|W^{H}H\right|}^{2}E_{s}}{\left\|W\right\|N_{0}}.$$

where *E_S_* is symbol energy, and *N*_0_ is noise power spectral density. Under this constraint, maximizing SNR becomes equivalent to minimizing the effective noise power $\left\|W\right\|N_{0}$. The SNR maximization under unit-norm constraint $\left\|W\right\|=1$ is formulated as

(9) $$\underset{W}{\max}\gamma =\frac{{\left|W^{H}H\right|}^{2}E_{s}}{N_{0}},\;s.t.\;{\left\|W\right\|}^{2}=1.$$

Using the Lagrange multiplier *λ*,

(10) $$L=W^{H}HHW^{H}W-\lambda (W^{H}W-1).$$

Taking gradient w.r.t. **W**,

(11) $$\nabla wL=2HH^{H}W-2\lambda W=0.$$

Thus, the maximum received SNR is denoted as

(12) $$SNR=\frac{\left\|H\right\|E_{s}}{N_{0}}.$$

where the channel gain is $g=\left\|H\right\|$.

**Figure 9:**
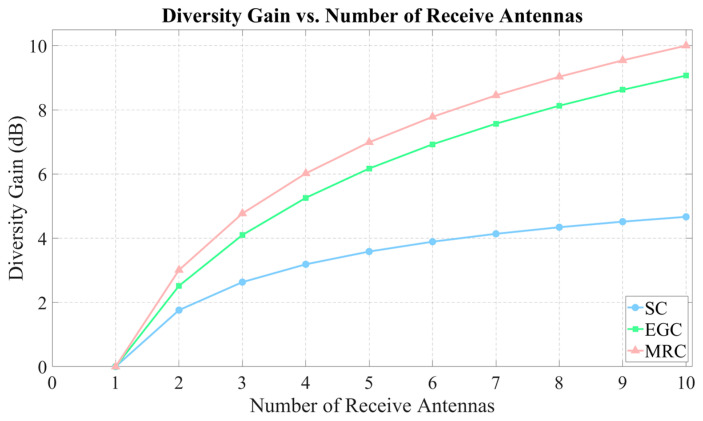
Diversity reception gain vs. number of receiver antennas diagram.

We compared systems transmitting identical symbols across all paths to exploit diversity gain through signal combining at the receiver. As shown in Figure 9, in a single-transmitter antenna system, MRC provides superior gain compared to Selection Combining (SC) and Equal Gain Combining (EGC). In our commonly deployed 2 × 2 MIMO systems, employing MRC technology can achieve up to 9 dB of diversity gain [89,90].

By combining PDM with a MIMO architecture, signals transmitted on two orthogonal polarizations preserve inter-channel incoherence. When identical signals are transmitted over both channels, MRC can be applied at the receiver to achieve diversity gain. This approach not only prevents the disruption of coherence caused by phase differences induced by wavelength discrepancies during transmission but also simultaneously obtains diversity gain.

**Figure 10:**
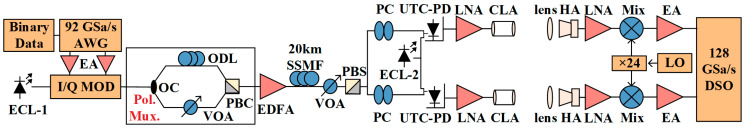
MIMO terahertz transmission system using PDM and MRC; VOA: variable optical attenuator; PBC: polarization beam combiner; Pol.Mux.: polarization multiplexer; CLA: cylindrical lens antenna [90].

In reference [91], we effectively resolved coherence disruption and enhanced performance in a 300 GHz terahertz wireless link over 200 m using a MIMO-PDM transmitter architecture combined with a MIMO-MRC receiver architecture. The system configuration is depicted in Figure 10. At the transmitter, PDM generates two orthogonally polarized 300 GHz terahertz signals (via a PDM module and dual UTC-PD opto-terahertz conversion), emitted by a high-gain cylindrical lens antenna array. At the receiver, dual antennas capture these independent polarization signals, with the MRC algorithm applied for optimal signal combining. This architecture innovatively leverages orthogonal polarization to ensure signal incoherence between paths, fundamentally preventing coherence disruption caused by phase differences in same-frequency/same-polarization signals, while enabling effective MRC implementation. Experimental results demonstrate successful achievement of a total data rate of 100 Gbit/s (50 Gbaud PDM-QPSK) over a 200 m wireless distance. The key performance improvement lies in the following: compared to single-channel transmission, this 2 × 2 MIMO-PDM-with-MRC solution achieves up to 1.2 dB SNR gain while successfully overcoming coherence disruption phenomena prevalent in long-distance MIMO transmission. This represents the first breakthrough solution simultaneously delivering performance gains and effective coherence disruption suppression in photonics-assisted terahertz MIMO systems.

### 3.3. High-Gain Antennas and Lenses

THz waves combine the advantages of millimeter waves and optical waves. Compared to millimeter waves, they offer broader usable frequency bands and superior beam directivity; they also provide enhanced security and superior anti-interference performance. Relative to optical waves, they are more efficient, exhibit stronger penetration capabilities, and demonstrate greater adaptability to harsh environments [92,93].

The main types of THz waveguides include conventional metallic rectangular waveguides, dielectric fiber waveguides, novel THz photonic crystal waveguides, surface plasmon polariton waveguides, and emerging two-dimensional material-based waveguides. The metallic rectangular waveguide, with its advantages of low dispersion and high power capacity, is employed in our experimental system for connecting with Cassegrain antennas [94,95,96]. THz antennas are indispensable devices in terahertz wireless communication systems for radiating and detecting terahertz waves. Driven by the unique properties of terahertz waves, key performance metrics of these antennas—particularly their broad operating bandwidth and gain—critically impact the overall system quality [97,98].

Antenna gain typically refers to the gain in the direction of maximum radiation. It can be expressed as

(13) $$G=\frac{4\pi A_{e}}{\lambda^{2}}.$$

where *A_e_* is the antenna effective aperture area and *λ = c/f* is the signal wavelength. As the frequency *f* increases to 0.1–10 THz, *λ* becomes significantly shorter than millimeter wavelengths. For a fixed physical aperture, THz antenna gain improves by >10× over mmWave systems, making aperture efficiency critical. Enhancing effective radiation area requires metasurfaces or diffractive optics at THz frequencies. Path loss in long-distance THz communication systems is a dominant attenuation factor modeled as follows [38,99]:

(14) $$P_{L}=20\log_{10}\left(\frac{4\pi df}{c}\right)+\alpha_{gas}d.$$

where *d* is the wireless transmission distance and *α_gas_* is the molecular absorption loss. In the THz frequency band, absorption peaks of water vapor and oxygen cause additional attenuation, increasing *α_gas_*. Thus, THz base station antennas require extremely high gain to overcome this enhanced path loss and improve system performance. While HAs serve as a low-cost, high-performance reference prototype—achieving up to 25 dBi gain with a simple structure—they prove insufficient for long-range THz wireless systems operating in complex environments [100]. Consequently, dual-reflector Cassegrain antennas (CAs) are widely adopted in THz systems.

**Figure 11:**
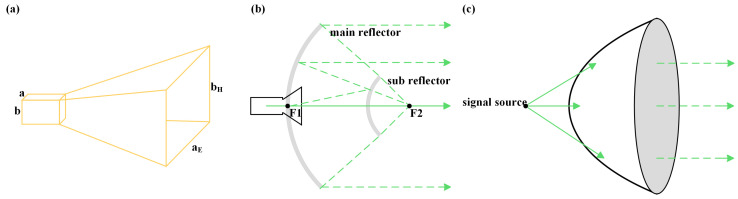
Terahertz antenna structure diagram: (**a**) HA; (**b**) CA; (**c**) lens antenna.

Figure 11 illustrates schematic diagrams of a rectangular HA, CA and lens antenna. The horn antenna features an intuitive and classic design—essentially a flared waveguide opening that gradually expands to form a trumpet-shaped radiating aperture. Signals feed into the narrow end of the waveguide; as electromagnetic waves propagate through the expanding horn and reach the broad aperture, the beam widens and shapes into a directional pattern. Here, a and b denote the width and height of the rectangular waveguide, while a_E_ and b_H_ represent the aperture side lengths in the E-plane and H-plane, respectively; R is the distance between the upper and lower horn apertures [101].

In contrast, the Cassegrain antenna employs a sophisticated dual-reflector system. Signals radiate from the feed source, first illuminating the sub-reflector. The sub-reflector redirects the signals to the primary reflector, which then collimates the beam for long-distance transmission. This structure “folds” the electromagnetic path via the sub-reflector, positioning the feed near the vertex of the primary reflector (or behind it) rather than at the distant focal point. This shortens the antenna′s physical length (effective focal length ≥ 2× physical length), reduces transmission loss, and maintains high gain and superior directivity. The sub-reflector effectively “relocates” the feed′s phase center to the focus of the parabolic surface [102].

The most prevalent antenna in our long-range THz transmission system is the THz lens antenna. Leveraging its focusing and imaging capabilities, this antenna enhances performance by achieving excellent directivity and high gain. Our lens adopts a plano-convex structure—simple in design and mature in manufacturing—optimized via geometric optics principles. Constructed from polytetrafluoroethylene (PTFE), it utilizes THz-wave refraction to collimate divergent radiation from the feed into directional narrow beams. PTFE is the ideal material for high-performance THz lens antennas, offering unmatched ultra-low loss and a low dielectric constant in THz bands; its refractive index is 1.4 in a wide wavelength range [103]. In long-range THz communication experiments, the lens gain at both the transmitter and receiver reaches 70 dB.

**Figure 12:**
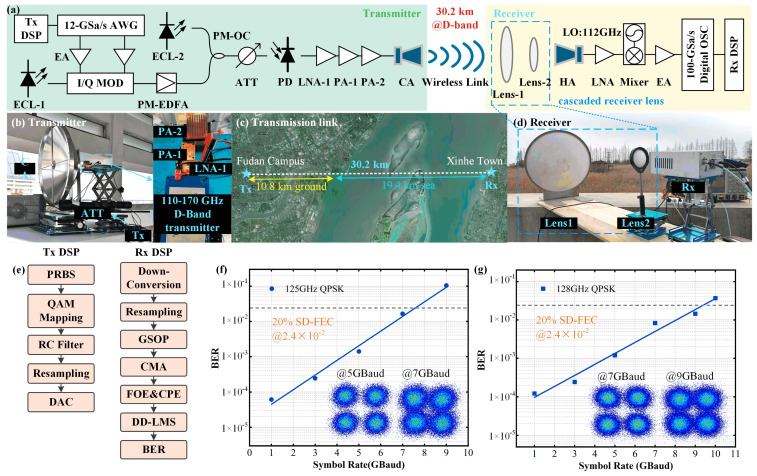
Ultra-long-distance terahertz wireless transmission system enabled by fusion of multiple antennas: (**a**) experimental setup of the ultra-long-distance D-band transmission system over 30.2 km; (**b**) photo of the transmitter; (**c**) photo of the transmission link; (**d**) photo of the receiver; (**e**) DSP structure at the transmitter side and receiver side; (**f**) BER curve of 125 GHz QPSK signal; and (**g**) BER curve of 128 GHz QPSK signal.

In the experimental setup diagram shown in Figure 12, to address the impact of Earth′s curvature on the transmission system, our transmitter is located atop the Guanghua Tower in Fudan University′s Handan Campus, while the receiver is situated at an open coastal area. by integrating a Cassegrain antenna (gain: 58 dBi, diameter: 60 cm), a dual-cascaded lens antenna (gain: 40 dB + 6 dB, diameter: 60 cm and 10 cm), and a horn antenna (gain: 25 dBi), we constructed a high-performance D-band wireless link. We use the Friis transmission equation for link power budget analysis. The transmit power is 23 dBm, the transmit antenna gain is 58 dBi, the signal frequencies are 125 GHz and 128 GHz, the transmission distance is 30.2 km, and the atmospheric losses are 28 dB at 125 GHz and 27.6 dB at 128 GHz, respectively. The received signal powers measure −40 dBm at 125 GHz and −39.8 dBm at 128 GHz. This system successfully achieved stable transmission of an 18 Gbps signal over 30.2 km (comprising 10.8 km terrestrial and 19.4 km sea-surface paths) under complex rain/fog conditions, establishing a new record rate-distance product of 543.6 Gbps·km. The integrated antenna system significantly enhanced signal transceiving capability, with the innovative dual-lens design at the receiver contributing an additional 6 dB gain compared to single-lens solutions, effectively overcoming the substantial path loss induced by ultra-long distances and severe weather.

### 3.4. Power Amplifiers

In THz systems, PAs serve as the core engine for overcoming path loss in ultra-high-frequency bands. According to the Friis transmission equation, the PA’s output power (P_t_) and antenna gain jointly determine the available link budget. Notably, the path loss term in the Friis equation scales inversely with the square of the distance d and the square of the wavelength λ. Given the extremely short wavelength of THz waves, path loss over the same distance is drastically higher than in microwave bands. A 1 mW PA can support a 10 m link in the 1 THz band [104]. Without a PA providing sufficient transmit power, the received signal would be entirely buried in noise, rendering the system inoperable. Thus, PAs are essential for counteracting extreme free-space loss and atmospheric attenuation during THz signal propagation and are critical for compensating the gain limitations of passive components like antennas.

**Figure 13:**
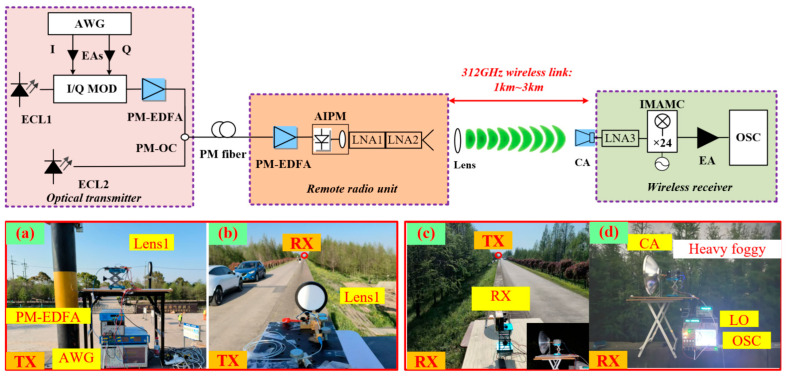
Complex environment transmission system based on InP semiconductor amplifier: (**a**) transmitter setup; (**b**) photo of transmitter; (**c**) photo of receiver; (**d**) receiver setup.

The PA’s output power, efficiency, and linearity directly dictate the feasible transmission distance, communication quality, and system power consumption, often representing the primary bottleneck in THz implementations. Common amplifiers in our experiments include PAs, LNAs, and InP semiconductor amplifiers [105,106,107]. Their core function is to deliver significant power gain, requiring both voltage amplification and robust current-driving capability to achieve high output power. However, when input power exceeds a threshold, gain compression occurs, eventually leading to output power saturation. In reference [88], the gain and noise figure of the PA and LNA are illustrated; in reference [108], a total amplifier gain of 99 dB was achieved for a 30 km ultra-long-range THz transmission system. Furthermore, we recently demonstrated a 3000 m wireless link at 312 GHz using an InP semiconductor amplifier—marking the first kilometer-range transmission reported above 300 GHz. As shown in Figure 13 of our experimental results, leveraging an InP semiconductor amplifier enabled kilometer-range transmission of terahertz waves under foggy conditions.

### 3.5. Heterodyne Coherent Detection Architecture

**Figure 14:**
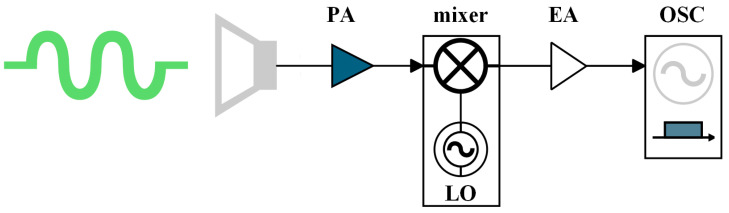
Heterodyne coherent detection schematic diagram.

Signal detection in terahertz communication systems is divided into two main schemes: direct detection and heterodyne detection. The direct detection scheme amplifies THz signals received by antennas through EAs and then directly down-converts the high-frequency signal to baseband using an envelope detector. It has a simple structure and low cost, and it is insensitive to phase noise, but it only supports demodulation of intensity-modulated signals and suffers from nonlinear distortion and power wastage, leading to limited signal quality, making it suitable for low-speed, low-cost scenarios [109,110]. Therefore, in long-range wireless terahertz communication systems, we prefer the heterodyne detection approach. The structure of the heterodyne detection scheme in THz communication is shown in Figure 14. This scheme primarily mixes an LO signal with the received signal to generate an IF signal. After digital down-conversion and capture by a digital oscilloscope, phase correction via DSP fully recovers the I/Q components of the vector-modulated signal [111].

The vector signal received by the THz receiver can be expressed as

(15) $$S(t)=A[I(t)\sin(2\pi f_{c}t+\theta_{c}(t))+Q(t)\cos(2\pi f_{c}t+\theta_{c}(t))].$$

Here, *A*, *f_c_*, and *θ_c_*(t) denote the amplitude, frequency, and phase of the THz signal, respectively. I(t) and Q(t) represent the in-phase (I) and quadrature (Q) components of the vector signal carried by the millimeter wave. The RF LO signal can be expressed as

(16) $$S_{LO}(t)=A_{LO}\cos(2\pi f_{LO}t+\theta_{LO}(t)).$$

where *A_LO_*, *f_LO_*, and *θ_LO_* represent the amplitude, frequency and phase of the RF LO signal, respectively. The THz signal is down-converted to a lower IF bandwidth via mixing with the LO signal, facilitating subsequent signal conditioning. The resulting IF signal after mixing is

(17) $$S_{IF}(t)=-jA_{LO}A[I(t)+jQ(t)]\exp(j2\pi f_{IF}t+j\theta_{IF}(t)).$$

The final output is obtained after digital down-conversion processing:

(18) $$\mathrm{Output}=j\cdot (-j)A_{LO}A[I(t)+jQ(t)]=A_{LO}A[I(t)+jQ(t)].$$

Although this scheme requires an LO and mixer—leading to increased system complexity and cost—it supports high-order modulation formats and enables distortion cancellation through DSP algorithms, significantly enhancing receiver sensitivity and spectral efficiency. Consequently, it serves as a core technology for long-distance terahertz communications.

## 4. High-Sensitivity SIMO Space–Ground Communication Architecture

With technological advancements and the integration of satellite communications, the internet, and mobile wireless networks, the Space–Terrestrial Integrated Network (STIN) has emerged as a strategic framework [112,113]. Satellite–ground communication constitutes the core of STIN, driven by three fundamental requirements: global coverage, capability enhancement, and service evolution.

While microwave bands remain the most widely used spectrum for satellite communications, they suffer from inherent limitations—namely, broad beamwidth and high power consumption—making them unsuitable for high-rate, large-bandwidth transmissions over long distances. FSO communication employs near-infrared optical carriers to establish terrestrial links within Earth′s atmosphere, but its long-range performance is severely constrained by atmospheric absorption, scattering, and turbulence, rendering it more suitable for inter-satellite links than satellite-to-ground scenarios demanding high data rates [114,115].

THz wireless communication emerges as a promising alternative to FSO for satellite-to-ground applications. Operating in the 0.1–10 THz range, it offers superior environmental robustness while preserving advantages of FSO-like bandwidth capacity [116,117,118].

### 4.1. Communication Models and System Designs for Terahertz Satellite–Ground Links

For the ultra-long-distance communication scenario between a geostationary orbit satellite (Fengyun-4) and a ground station (Shanghai), we demonstrated 100 Gbps high-speed simulated transmission over 36,000 km in the 300 GHz frequency band with 16QAM modulation through system-level simulation. System parameters are detailed in Table 1. Based on the Friis equation, the received signal power at the ground station is approximately −52.1 dBm. The receiver employs a 25 dB gain THz LNA followed by a 30 dB gain PA. After down-conversion (with 10 dB insertion loss), the signal passes through an additional 30 dB gain LNA. Accounting for the receiver’s 10 dB noise figure, the final signal power before demodulation reaches ~13 dBm.

**Figure 15:**
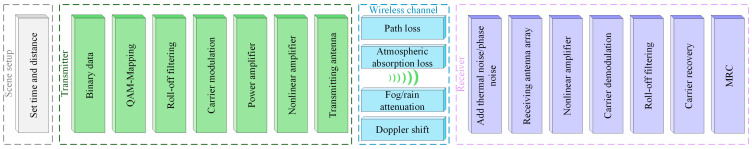
SIMO satellite–ground communication flowchart.

To enable higher data rates, we implement the MIMO-MRC technique described in Section 3.2, replacing a single 4.5 m large-aperture antenna with four smaller-aperture antennas. With four receiver antennas, MRC combining theoretically quadruples the SNR (6 dB gain). Equivalent aperture calculations show each smaller antenna requires a diameter of 2.06 m. After diversity reception, advanced DSP techniques—including carrier demodulation, matched filtering, carrier recovery, and MRC—are applied. The system’s workflow and schematic are illustrated in Figure 15.

### 4.2. Simulation Results and Discussion

**Figure 16:**
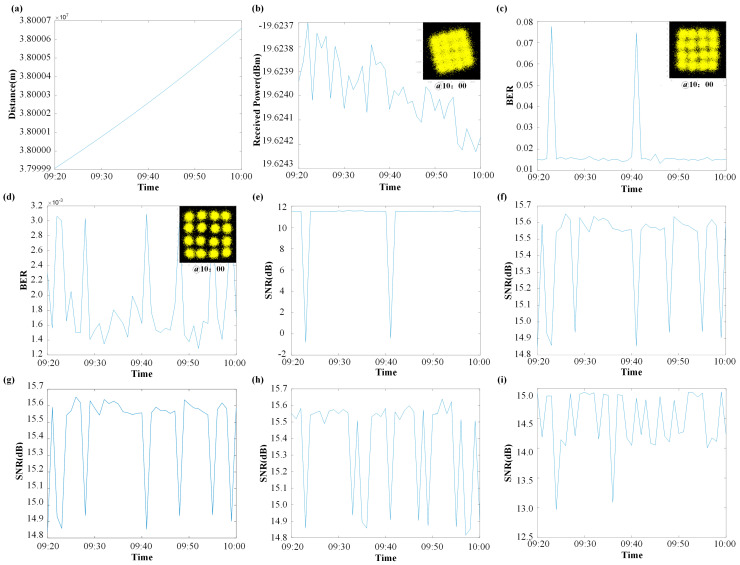
Simulation results of high-sensitivity SIMO satellite–ground communication architecture: (**a**) time vs. communication distance; (**b**) time vs. received power; (**c**) BER before diversity combining; (**d**) BER after diversity combining; (**e**) SNR before diversity combining; (**f**) SNR after diversity combining; (**g**) SNR under clear sky conditions; (**h**) SNR under fog attenuation; (**i**) SNR under rain attenuation.

This Earth–satellite communication simulation under the SIMO architecture successfully modeled the dynamic satellite–ground station distance variation over 40 min caused by Earth′s revolution and rotation, along with its impact on communication performance, as shown in Figure 16a,b. The distance increased from 3.79999 × 10^7^ m to 3.80007 × 10^7^ m, exhibiting a significant decline in P_r_ with increasing range.

As shown in Figure 16, the simulation results robustly validate the effectiveness of the core implemented technologies: DSP and MRC diversity reception techniques substantially enhanced communication quality. At the receiver, the constellation diagram effectively restored phase alignment after DSP processing and demonstrated sharper, more distinct clustering characteristics following MRC combining. The BER prior to diversity reception (Figure 16c) fluctuated around the 10^−2^ level, while post MRC combining (Figure 16d), it significantly reduced to the 10^−3^ order of magnitude. Concurrently, a system gain of approximately 4 dB in SNR was achieved.

Addressing weather-induced attenuation in the satellite-to-Earth link, the simulation contrasted performance under clear sky, fog attenuation, and rain attenuation conditions. Under fog attenuation (Figure 16h), SNR fluctuations remained below 0.1 dB compared to the clear sky baseline (Figure 16g). Rain attenuation caused more noticeable SNR degradation, though the maximum attenuation was controlled at approximately 1 dB.

This simulation not only demonstrates the technical feasibility of utilizing the terahertz band for Earth–satellite communications under dynamic link conditions and typical weather scenarios but also quantitatively proves the critical role of DSP and diversity reception techniques in enhancing link robustness and reliability.

## 5. Conclusions

This paper summarizes our advances in long-distance terahertz communication, systematically tackling the ‘dual attenuation dilemma′ of path loss and atmospheric attenuation through co-designed photonic–electronic architectures and adaptive signal processing. By developing hybrid THz generation schemes—including photonically assisted probabilistic 64QAM achieving a record 1.0488 Tbps throughput in the 330–500 GHz band—we established seamless FiWi integration capable of bridging fiber and Tbps wireless links. To overcome severe path loss exceeding 120 dB/km, our multi-pronged approach synergizes high-gain antenna–lens systems, InP semiconductor amplifiers enabling the first 3 km fog-penetrating link at 312 GHz, and modulation-robust DSP algorithms like MIMO-VNE. These innovations culminated in a landmark 30.2 km D-band transmission with 543.6 Gbps·km capacity–distance product, representing an 8-fold improvement over prior records. For dynamic channel adaptability, the proposed MIMO-MRC architecture demonstrated 9 dB diversity gain through polarization-multiplexed transmission and coherent combining, while real-time VQ-VAE-based processing maintained a BER below 3.4 × 10^−2^ under orbital dynamics and weather impairments. Critically, our satellite–ground simulation validated THz viability for space–terrestrial integration, achieving 100 Gbps over 36,000 km at 300 GHz with MRC diversity reducing the BER from the order of 10^−2^ to 10^−3^. Collectively, these breakthroughs provide a scalable framework for 6G integrated SAG networks, delivering three foundational capabilities: terabit-per second-capable ultra-high-capacity backhaul via fiber–THz convergence; robust long-haul connectivity across distances reaching 30 km over land and extending to 36,000 km for space links, enabled through photonic–electronic co-optimization; and dynamic channel resilience enabled by intelligent MIMO-DSP adaptation. Future work will extend to non-line-of-sight scenarios using reconfigurable intelligent surfaces and standardization pathways for THz-enabled 6G deployments.

## Figures and Tables

**Table 1:** High-sensitivity SIMO communication architecture system parameters.

Parameter	Value
Frequency	300 GHz
Distance	36,000 km
Symbol Rate	25 GBaud
Symbol Length	32,768
Sampling Points per Symbol	30
Modulation Order	16
TX Antenna Diameter	4.5 m
RX Antenna Diameter	2.06 m
Aperture Efficiency	0.6
Number of RX Antennas	4
Transmit Power	1 W

## Data Availability

The data presented in this study is available on request from the corresponding author. The data is not publicly available due to privacy restrictions.
